# *Psychrobacter sanguinis* Wound Infection Associated with Marine Environment Exposure, Washington, USA

**DOI:** 10.3201/eid2410.171821

**Published:** 2018-10

**Authors:** Jesse Bonwitt, Michael Tran, Angela Droz, Anna Gonzalez, William A. Glover

**Affiliations:** Centers for Disease Control and Prevention, Atlanta, Georgia, USA (J. Bonwitt);; Durham University, Durham, England (J. Bonwitt);; Washington State Department of Health, Shoreline, Washington, USA (J. Bonwitt, M. Tran, W.A. Glover);; Harrison Medical Center, Bainbridge Island, Washington, USA (A. Droz);; Kitsap Public Health District, Bremerton, Washington, USA (A. Gonzalez)

**Keywords:** cellulitis, *Psychrobacter*, *Psychrobacter sanguinis*, RNA, ribosomal, rRNA, 16S, northwestern United States, Pacific Ocean, Puget Sound, Pacific Northwest Coast, bacteria, marine environment, Washington, USA, exposure, wound infection, fishing, opportunistic infection, antimicrobial drugs, bacteria culture, United States

## Abstract

We report a 26-year-old man with *Psychrobacter sanguinis* cellulitis of a wound sustained during ocean fishing in Washington, USA, in 2017. *Psychrobacter* spp. are opportunistic pathogens found in a wide range of environments. Clinicians should be aware of *Psychrobacter* spp. and perform 16S rRNA sequencing if this pathogen is suspected.

In February 2017, a 26-year-old man sought treatment at an urgent care facility (Harrison Medical Center, Bainbridge Island, Washington, USA) for a hand laceration and reported tingling in his fingers, hand, and forearm. The laceration was a healing 6-cm diagonal cut across the dorsum of the hand with surrounding erythema and cellulitis without active bleeding. Vital signs (blood pressure, pulse, respiration, and temperature) were within reference ranges. The patient reported that his wound occurred while he was cutting squid bait when he was ocean fishing for crabs in Puget Sound (Pacific Ocean), Washington, USA. The squid bait was purchased frozen from an independent fishing retail store. The patient did not report any other pertinent medical history (e.g., immunosuppression). We cleaned and dressed his wound, administered tetanus vaccine (Adacel; Sanofi Pasteur Inc., Swiftwater, Pennsylvania, USA) prophylactically, and treated him as an outpatient with oral cephalexin and topical bacitracin zinc. He fully recovered.

We submitted a wound swab sample to the hospital laboratory for bacterial culture. The cultures yielded rare colonies of coagulase-negative *Staphylococcus* spp. and light growth of a gram-negative rod. In a subsequent attempt to identify the unknown gram-negative rod by VITEK 2 (bioMérieux Inc., Durham, NC, USA), the results suggested *Brucella* spp. The isolate was sent to Washington State Public Health Laboratories (Shoreline, Washington, USA) for confirmatory testing, but the isolate tested negative for *Brucella* spp. by PCR. No leftover squid bait was available for sampling. Gram staining of the isolate revealed gram-negative coccobacilli arranged in pairs with rare cells that retained crystal violet stain. When culturing at 35°C was performed, medium-sized, convex, sticky, nonhemolytic colonies formed on blood agar and pinpoint colonies with pitting on chocolate agar. Colonies were catalase, oxidase, and urease positive. The isolate could not be identified by matrix-assisted laser desorption/ionization mass spectrometry (MALDI Biotyper CA System, research-use-only version 4.1.8; Bruker Daltonics Inc., MA, USA). Sequencing of 16S rRNA performed by the Centers for Disease Control and Prevention (Atlanta, Georgia, USA) identified the bacterium as *Psychrobacter sanguinis* (GenBank accession no. MH178035).

We performed antimicrobial susceptibility testing under aerobic conditions at 35°C using disk diffusion testing. Despite the absence of standardized break points for *Psychrobacter* spp., the large zone sizes indicated that the isolate was susceptible to cefazolin, cefepime, cefoxitin, ceftriaxone, ciprofloxacin, meropenem, penicillin, and tetracycline. The isolate tested negative for β-lactamase.

*Psychrobacter* are psychrotrophic (i.e., cold tolerant), gram-negative bacteria of the family *Moraxellaceae* (*1*). *Psychrobacter* spp. have been isolated from marine species (crustaceans, fish, and marine mammals); marine environments (seabed and seaweed); food products (seafood, cheese, and meat); storks; pig digestive tracts; and lamb lungs (https://www.ncbi.nlm.nih.gov/Taxonomy/Browser/wwwtax.cgi). *Psychrobacter* spp. might also be a component of the human microbiota; studies have demonstrated the presence of *P. arenosus*, *P. faecalis*, *P. phenylpyruvicus*, and *P. pulmonis* in the human gut (*2*).

Only a subset of *Psychrobacter* spp. are considered medically relevant opportunistic pathogens on the basis of a limited number of published case reports (*1*,*3*). Clinical manifestations depend on the infection site and include bacteremia (*4*,*5*), meningitis (*6*,*7*), surgical wound infection (*8*), and ocular infection (*9*). Of these cases, only 1 was associated with exposure to a marine environment; in that case, the patient experienced *P. phenylpyruvicus* bacteremia after consuming a raw geoduck clam that was possibly imported from the Pacific Northwest (*5*).

*P. sanguinis* was reported as a new species in 2012, after retrospective isolation from the blood of 4 patients in New York, USA (*10*). *P. sanguinis* infection was subsequently reported in a patient with meningitis in France (*7*), and an organism closely related to *P. sanguinis* (98% identity of 16S rRNA) was reported in a patient with meningitis in Mexico (*6*). One of these patients acquired the infection nosocomially, but the source of the infections could not be determined, and exposure to marine environments was not reported for either case. Both patients were treated with antimicrobial drugs; 1 patient fully recovered, and the other died from complications, including septic shock. *P. sanguinis* has previously been described as broadly susceptible (*7*); however, *Psychrobacter* spp. have displayed penicillin resistance (*1*).

We describe a case of *P. sanguinis* infection in a healthy person after wound exposure to squid bait and seawater of the Pacific Northwest Coast. The source of the infection could not be determined, but isolation of *Psychrobacter* spp. from a wide range of environments suggests the infection could have occurred from exposure to the marine environment. Contamination of the wound by human gut microbiota cannot be excluded but is unlikely, given that only 2 types of bacteria were isolated from the wound. The wound displayed cellulitis, a presentation consistent with infection by an opportunistic pathogen; this finding, therefore, expands the clinical spectrum of *P. sanguinis* infection. Clinicians and laboratorians should be aware of the opportunistic potential of *Psychrobacter* spp. and the limitations of commercial identification systems for confirming these agents.
